# Association between Kidney Function and Framingham Global Cardiovascular Disease Risk Score: A Chinese Longitudinal Study

**DOI:** 10.1371/journal.pone.0086082

**Published:** 2014-01-20

**Authors:** Bo Jin, Xiaojuan Bai, Lulu Han, Jing Liu, Weiguang Zhang, Xiangmei Chen

**Affiliations:** 1 Department of Gerontology and Geriatrics, Shengjing Hospital of China Medical University, Shenyang, China; 2 Department of Circulation, Asia Heart Hospital, Wuhan, China; 3 Department of Kidney, General Hospital of Chinese People’s Liberation Army, Beijing, China; University of KwaZulu-Natal, South Africa

## Abstract

**Background:**

Chronic kidney disease (CKD) is generally considered an independent risk factor for cardiovascular disease (CVD) development, but rates in individuals with estimated glomerular filtration rate (eGFR) >60 ml/min/1.73 m^2^ are uncertain. The Framingham global CVD risk score (FRS) equation is a widely accepted tool used to predict CVD risk in the general population. The purpose of the present study was to examine whether an association exists between eGFR and FRS in a Chinese population with no CKD or CVD.

**Methods:**

A total of 333 participants were divided into three groups based on FRS. The Chronic Kidney Disease Epidemiology Collaboration (CKD-EPI) equation and CKD-EPI equation for Asians (CKD-EPI-ASIA) were used to measure eGFR.

**Results:**

A significant inverse association between eGFR and FRS was confirmed with Pearson correlation coefficients of –0.669, –0.698 (eGFR_CKD-EPI_, *P*<0.01) and –0.658, –0.690 (eGFR_CKD-EPI-ASIA_, *P*<0.01). This association gradually diminished with progression from the low- to high-risk groups (eGFR_CKD-EPI_, *r* = –0.615, –0.282, –0.197, *P*<0.01, *P*<0.01, *P*>0.05; similar results according to the CKD-EPI-ASIA equation). In the low- or moderate-risk new-groups, this association became stronger with increased FRS (eGFR_CKD-EPI-ASIA_, *r* = –0557, –0.622 or –0.326, –0.329, *P*<0.01). In contrast to the results from 2008, eGFR was independently associated with FRS following adjustment for traditional cardiovascular risk factors (*P*<0.05).

**Conclusion:**

Renal function has multiple influences on predicting CVD risk in various populations. With increasing FRS and decreasing eGFR, it is also independently associated with CVD, even in individuals with eGFR >60 ml/min/1.73 m^2^.

## Introduction

Cardiovascular diseases (CVD), including coronary heart disease, stroke, peripheral artery disease and heart failure, constitute major public health issues worldwide [1]. Lifetime risk of CVD is substantial, and the condition is often silent or may occur without warning, underscoring the importance of prevention [2]. Efforts to estimate absolute CVD risk of individuals have devised numerous risk prediction tools that synthesize vascular risk factors, such as the Framingham global CVD risk score (FRS) equation. In addition to traditional cardiovascular risk factors, such as age, sex, high blood pressure, smoking, dyslipidemia and diabetes, investigators are trying to detect more markers associated with CVD, including those in which renal function is considered.

Most nephrologists agree that estimated glomerular filtration rate (eGFR) is the most feasible clinical measure of renal function. Many studies have demonstrated that reduced eGFR is a predictor of major cardiovascular events [3]–[6]. However, the level of eGFR at which increased risk of CVD becomes apparent remains uncertain. Several studies have suggested that even small reductions in eGFR within the apparently normal range are associated with increased risk of CVD, while others suggest that the increase in risk may not become apparent until eGFR declines to <60 ml/min/1.73 m^2^ [7]. The present study was performed in a Chinese community-based population with eGFR >60 ml/min/1.73 m^2^ and with no CVD to examine whether an association exists between eGFR and FRS.

## Materials and Methods

### 1. Study Participants

This community-based longitudinal study was begun with the enrollment of subjects in a study that was approved by the Ethics Committee of China Medical University in Shenyang in 2008 with follow-up conducted in 2011. All study subjects signed informed consent forms. In 2008, 501 healthy subjects were confirmed to be study participants out of 1500 volunteers following examinations, which included measurements of fasting blood glucose (FBG), triglyceride (TG), total cholesterol (TC), high-density lipoprotein cholesterol (HDL-C), low-density lipoprotein cholesterol (LDL-C), serum creatinine (Scr), blood urea nitrogen (BUN), and blood uric acid (UA). Electrocardiograms and chest X-rays were also performed. Following a three-year follow-up, 124 subjects were lost. As a result, 377 study subjects received the same examinations as were performed in 2008. Ultimately to enforce self-control, 333 subjects were confirmed as participants after excluding 34 subjects for data loss and 10 subjects with eGFR values <60 ml/min/1.73 m^2^.

### 2. Data Collection

Participants who self-reported that they smoked regularly during the previous 12 months prior to enrollment were classified as smokers [2], while the others were classified as non-smokers. Height, weight, waist circumference and hip circumference were measured at baseline, and body mass index (BMI), body surface area (BSA) and waist-to-hip ratio (WHR) were calculated. Blood pressure measurements were performed on the left arm of seated participants with a mercury-column sphygmomanometer and an appropriately sized cuff; an average of two physician-obtained measures constituted the examination of blood pressure. Hypertension was defined as a systolic blood pressure (SBP) ≥140 mmHg or diastolic blood pressure (DBP) ≥90 mmHg and/or taking antihypertensive medications. Diabetes was defined as fasting plasma glucose ≥126 mg/dl or the use of insulin or oral hypoglycemic medications. All biochemical indicators were tested using a Hitachi 747 automatic biochemistry analyzer following a 12 h fast.

### 3. Framingham Risk Score Criterion [2] and Grouping

The Framingham global CVD risk score equation was used to calculate FRS according to cardiovascular risk factors of study participants. This equation includes six risk factors: age, systolic blood pressure, history of diabetes mellitus, cigarette smoking status, HDL-C and TC/LDL-C level [2]. Each risk factor of this equation has its corresponding Framingham score. The Framingham score for non-smokers was 0 points while that for male and female smokers was 4 points and 3 points, respectively; the score for participants with no diabetes history was 0 points; and the score for male and female diabetes mellitus patients was 3 points and 4 points, respectively. FRS is the sum of these six risk factor points. Participants were divided into three groups according to FRS [2]: low risk group: FRS ≤7 points in males and ≤9 points in females, with 0% to 6% risk; moderate risk group: FRS of 8–14 points in males and 10–17 points in females, with 6% to 20% risk; high risk group: FRS ≥15 points in males and ≥18 points in females, with >20% risk.

### 4. Estimated Glomerular Filtration Rate (eGFR) Calculations

The Chronic Kidney Disease Epidemiology Collaboration (CKD-EPI) equation was developed in 2009. Compared with the widely-used Modification of Diet in Renal Disease (MDRD) study equation, the CKD-EPI equation showed higher accuracy among individuals, especially at eGFR >60 ml/min/1.73 m^2^ [8]. Recently, a study demonstrated that the CKD-EPI equation estimated a reasonable distribution of eGFR compared with the MDRD equation in healthy Chinese adult populations [9]. Since creatinine generation differs among ethnic groups, the CKD-EPI-ASIA equation was developed to allow more accurate estimates in Asian populations [10].

#### 4.1. Chronic Kidney Disease Epidemiology Collaboration (CKD-EPI) Equation [8]

Males:

Scr <0.9 mg/dl; eGFR_CKD-EPI_ = 141×(Scr/0.9)^−0.411^×0.993 ^Age^

Scr >0.9 mg/dl; eGFR_CKD-EPI_ = 141×(Scr/0.9) ^−1.209^×0.993 ^Age^

Females:

Scr <0.7 mg/dl; eGFR_CKD-EPI_ = 144×(Scr/0.7)^−0.329^×0.993 ^Age^

Scr >0.7 mg/dl; eGFR_CKD-EPI_ = 144×(Scr/0.7)^−1.209^×0.993 ^Age^

#### 4.2. Modified CKD-EPI Equation for Asians [10]

Males: eGFR_CKD-EPI-ASIA_ = eGFR_CKD-EPI_×1.057

Females: eGFR_CKD-EPI-ASIA_ = eGFR_CKD-EPI_×1.049

In the above equations, the units for each index were: age (years), Scr (mg/dl), and eGFR (ml/min/1.73 m^2^).

### 5. Statistical Analyses

The Kolmogorov-Smirnov test was used for normal distribution analyses. Continuous data are expressed as means ± SD and the differences in these variables among groups were examined using a one-way analysis of variance (ANOVA). Least-square difference (LSD) analyses were utilized for comparisons between pairs of groups. Paired samples t-test was used for self-controlling among individuals and risk groups. Categorical data are expressed as numbers (percentile) [*n* (%)], and the differences in these variables among groups were examined using Chi-square statistics. Pearson correlation coefficient was used to assess the association between FRS and eGFR. Multiple regression analyses were used to adjust for traditional cardiovascular risk factors, including age, sex, TC, SBP, HDL-C, smoking, and fasting blood glucose. *P-*values <0.05 were considered to indicate statistical significance. All statistical analyses were performed using SPSS 17.0 statistical software.

## Results

### 1. Study Characteristics

In 2008, there were a total of 333 participants aged 34–84 (56.0±12.5) years old, consisting of 138 males (41.4%) and 195 females (58.6%) (Table 1). Participants were divided into three groups according to their FRS: low risk group–153 persons (45.9%), consisting of 28 males (18.3%) and 125 females (81.7%); moderate risk group–150 persons (45.1%), consisting of 80 males (53.3%) and 70 females (46.7%); high-risk group–30 persons (9.0%), who were all males (100%). Age, BMI, BSA, SBP, DBP, TG, TC, LDL-C, BUN, Scr, UA and FBG gradually increased with progression from the low- to high-risk groups (*P*<0.01), HDL-C gradually decreased (*P*<0.01), and the proportion of smokers and male participants gradually increased (*P<*0.01). Age, BMI, BSA, WHR, SBP, DBP, TG, TC, HDL-C, LDL-C, BUN, Scr, UA, and FBG were significantly different between the low-risk and moderate-risk groups (*P<*0.05). Age, BMI, BSA, SBP, DBP, TC, HDL-C, LDL-C, BUN, Scr, UA and FBG were significantly different between the low-risk and high-risk groups (P<0.05). Age, BSA, SBP, Scr, and UA were significantly different between the moderate-risk and high-risk groups (P<0.05).

**Table 1:** Basic information of participants in different risk groups studied in 2008.

Characteristic	Total (n = 333)	Low risk group(n = 153)	Moderate risk group(n = 150)	High risk group(n = 30)	*P*
Age (years)	56.0±12.5	47.9±9.5	61.7±10.4a	69.4±6.9ab	<0.001
Male/Female(%)	138(41.4%)/195(58.6%)	28(18.3%)/125(81.7%)	80(53.3%)/71(46.7%)	30(100%)/0(0%)	<0.01
Smoker (%)	58 (17.4%)	11 (7.2%)	34 (22.7%)	13 (43.3%)	<0.01
BMI	23.6±3.0	22.9±3.0	24.0±2.8a	25.1±2.8a	<0.001
BSA	48.0±4.6	46.6±3.9	48.5±4.8a	52.3±3.4ab	<0.001
WHR	0.85±0.34	0.80±0.07	0.89±0.49a	0.87±0.06	0.071
SBP (mmHg)	123.7±13.0	114.9±10.9	130.3±9.8a	135.2±6.6ab	<0.001
DBP (mmHg)	75.4±9.1	70.7±8.5	79.4±7.4a	79.7±7.6a	<0.001
TG (mmol/L)	1.35±0.99	1.14±0.70	1.57±1.19a	1.29±0.87	0.001
TC (mmol/L)	5.21±1.06	4.93±0.93	5.46±1.12a	5.47±1.05a	<0.001
HDL-C (mmol/L)	1.65±0.40	1.72±0.39	1.60±0.41a	1.52±0.39a	0.007
LDL-C (mmol/L)	2.90±0.75	2.66±0.70	3.10±0.73a	3.09±0.79a	<0.001
BUN (mmol/L)	5.24±1.25	4.89±1.16	5.50±1.25a	5.75±1.23a	<0.001
Scr (µmol/L)	61.8±12.6	56.5±10.0	64.7±12.7a	74.3±10.4ab	<0.001
UA (µmol/L)	285.8±75.5	256.3±64.8	304.7±74.0a	341.2±73.9ab	<0.001
FBG (mmol/L)	5.52±0.45	5.43±0.38	5.60±0.47a	5.60±0.52a	0.002
FRS	8.8±5.4	4.0±3.5	12.2±1.9a	16.8±1.5ab	<0.001
eGFR_CKD-EPI_(ml/min/1.73 m^2^)	100.3±12.9	107.7±10.9	95.2±11.0a	88.2±9.6ab	<0.001
eGFR_CKD-EPI-ASIA_(ml/min/1.73 m^2^)	105.6±13.6	113.1±11.5	100.3±11.7a	93.2±10.2ab	<0.001

Smoking information and sex are expressed as *n* (%) and are tested by the Chi-square method; other parameters are expressed as *χ*±*s* and are tested by ANOVA. a: Compared with low risk group, *P*<0.05; b: compared with moderate risk group, *P*<0.05. BMI: body mass index; BSA: body surface area; WHR: waist-to-hip ratio; SBP: systolic blood pressure; DBP: diastolic blood pressure; TG: triglyceride; TC: total cholesterol; HDL-C: high-density lipoprotein cholesterol; LDL-C: low-density lipoprotein cholesterol; BUN: blood urea nitrogen; Scr: serum creatinine; UA: blood uric acid; FBG: fasting blood glucose. FRS: Framingham global CVD risk scores; eGFR_CKD-EPI_: estimated glomerular filtration rate calculated by Chronic Kidney Disease Epidemiology Collaboration (CKD-EPI) equation; eGFR_CKD-EPI-ASIA_: estimated glomerular filtration rate calculated by Modified CKD-EPI equation for Asians.

Compared with corresponding values obtained in 2008, values for age, BSA, SBP, DBP, LDL-C, UA and FBG obtained in 2011 were increased significantly in the entire study population (*P*<0.01) (Table 2), age, WHR, SBP, DBP, TC, LDL-C, UA and FBG increased significantly in the low-risk group (*P*<0.05), age, SBP, DBP, TG, TC, LDL-C, FBG increased significantly in the moderate-risk group (*P*<0.05), and age, BSA, SBP, DBP, TC, LDL-C increased significantly in the high-risk group (*P*<0.05), while HDL-C in all groups decreased significantly (*P*<0.01).

**Table 2:** Basic information of participants in different risk groups studied in 2011.

Characteristic	Total (n = 333)	Low risk group(n = 153)	Moderate risk group(n = 150)	High risk group(n = 30)	*P*
Age (years)	58.6±12.5*	50.6±9.7*	64.1±10.5a*	72.1±6.8ab*	<0.001
Male/Female(%)	138(41.4%)/195(58.6%)	28(18.3%)/125(81.7%)	80(53.3%)/70(46.7%)	30(100%)/0(0%)	<0.01
Smoking (%)	64 (19.2%)	12 (7.8%)	39 (26.0%)	13 (43.3%)	<0.01
BMI	23.6±3.2	23.0±3.1	24.0±3.2a	24.8±3.2a	0.001
BSA	47.7±4.7*	46.4±4.1	48.4±5.1a	51.2±3.4ab*	<0.001
WHR	0.84±0.08	0.82±0.06*	0.86±0.06a	0.91±0.15ab	<0.001
SBP (mmHg)	130.4±19.6*	120.0±15.1*	137.9±18.7a*	146.3±19.6ab*	<0.001
DBP (mmHg)	77.7±10.4*	72.4±8.7#	81.8±9.5a*	83.9±10.2a#	<0.001
TG (mmol/L)	1.24±0.99	1.21±1.24	1.31±0.74*	1.06±0.48	0.065
TC (mmol/L)	5.16±0.95	5.06±0.93#	5.29±0.97a#	5.07±0.90#	0.097
HDL-C (mmol/L)	1.47±0.35*	1.53±0.35*	1.42±0.34a*	1.36±0.36a*	0.006
LDL-C (mmol/L)	3.29±0.85*	3.14±0.84*	3.44±0.83a*	3.37±0.86#	0.008
BUN (mmol/L)	5.28±1.30	4.89±1.12	5.61±1.37a	5.56±1.32a	<0.001
Scr (µmol/L)	62.4±13.2	57.1±10.8	65.2±13.3a	75.1±11.2ab	<0.001
UA (µmol/L)	295.0±74.6*	268.5±65.1*	312.8±71.1a	341.0±89.7ab	<0.001
FBG (mmol/L)	5.21±0.51*	5.06±0.42*	5.32±0.55a*	5.41±0.46a	<0.001
FRS	10.6±5.6*	6.2±4.5*	13.7±2.9a*	18.0±2.1ab*	<0.001
eGFR_CKD-EPI_(ml/min/1.73 m^2^)	98.0±12.9*	105.2±11.3*	93.1±10.6a*	85.9±9.8ab	<0.001
eGFR_CKD-EPI-ASIA_(ml/min/1.73 m^2^)	103.1±13.5*	110.6±11.9*	98.0±11.3a*	90.8±10.4ab	<0.001

Smoking information and sex are expressed as *n* (%) and are tested by the Chi-square method; the other parameters are expressed as *χ*±*s* and are tested by ANOVA. a: Compared with low risk group in 2011, *P*<0.05; b: compared with moderate risk group in 2011, *P*<0.05.

* Compared with the same population in 2008, *P*<0.01;

# Compared with the same population in 2008, *P*<0.05.

BMI: body mass index; BSA: body surface area; WHR: waist-to-hip ratio; SBP: systolic blood pressure; DBP: diastolic blood pressure; TG: triglyceride; TC: total cholesterol; HDL-C: high-density lipoprotein cholesterol; LDL-C: low-density lipoprotein cholesterol; BUN: blood urea nitrogen; Scr: serum creatinine; UA: blood uric acid; FBG: fasting blood glucose. FRS: Framingham global CVD risk scores; eGFR_CKD-EPI_: estimated glomerular filtration rate calculated by Chronic Kidney Disease Epidemiology Collaboration (CKD-EPI) equation; eGFR_CKD-EPI-ASIA_: estimated glomerular filtration rate calculated by Modified CKD-EPI equation for Asians.

### 2. FRS and eGFR

#### 2.1. FRS in Different Risk Groups Calculated in 2008 and in 2011

In 2008, the mean FRS in the whole study population was 8.8±5.4 points, with a range of –3.0 points to 20.0 points, and FRS was significantly different among the three risk groups (*P*<0.001) (Table 1). Compared with the low-risk group, FRS in the other two groups was statistically increased (*P*<0.01), and compared with the moderate-risk group, FRS in the high-risk group was significantly increased (*P*<0.01).

In 2011, the mean FRS in the whole study population was 10.6±5.6 points, with a range of –3.0 points to 22.0 points (Table 2). Compared to themselves, FRS in the whole study population and each risk group (low-, moderate- and high-risk groups) increased significantly (*P*<0.01, *P*<0.01, *P*<0.01, *P*<0.01, respectively).

#### 2.2. eGFR in Different Risk Groups Calculated in 2008 and in 2011

In 2008, the mean eGFR_CKD-EPI_ in the entire study population was 100±13 ml/min/1.73 m^2^, with a range of 62.0 ml/min/1.73 m^2^ to 128 ml/min/1.73 m^2^; the mean eGFR_CKD-EPI-ASIA_ in the whole study population was 106±14 ml/min/1.73 m^2^, with a range of 65.0 ml/min/1.73 m^2^ to 135 ml/min/1.73 m^2^ (Table 1). There was a statistically significant difference in eGFR among the three risk groups (*P*<0.001). From the low- to high-risk group, eGFR_CKD-EPI_ and eGFR_CKD-EPI-ASIA_ both decreased gradually in 2008 (*P*<0.01). Compared with the low-risk group, eGFR_CKD-EPI_ and eGFR_CKD-EPI-ASIA_ in the two other risk groups were significantly lower (*P<*0.01). Compared with the moderate-risk group, eGFR_CKD-EPI_ and eGFR_CKD-EPI-ASIA_ in the high-risk group were significantly lower (*P*<0.01).

In 2011, the mean eGFR_CKD-EPI_ in the whole study population was 98.0±12.9 ml/min/1.73 m^2^, with a range of 61.4 ml/min/1.73 m^2^ to 129 ml/min/1.73 m^2^ and the mean eGFR_CKD-EPI-ASIA_ in the whole study population was 103±14 ml/min/1.73 m^2^, with a range of 64.9 ml/min/1.73 m^2^ to 136 ml/min/1.73 m^2^ (Table 2). Compared to themselves, eGFR_CKD-EPI_ in the whole study population calculated in 2008, in the low-risk and moderate-risk groups decreased significantly when recalculated in 2011 (*P*<0.001, *P*<0.01, *P*<0.01), and the same conclusion was determined from the CKD-EPI-ASIA equation.

### 3. The Association between FRS and eGFR

**Figure 1:**
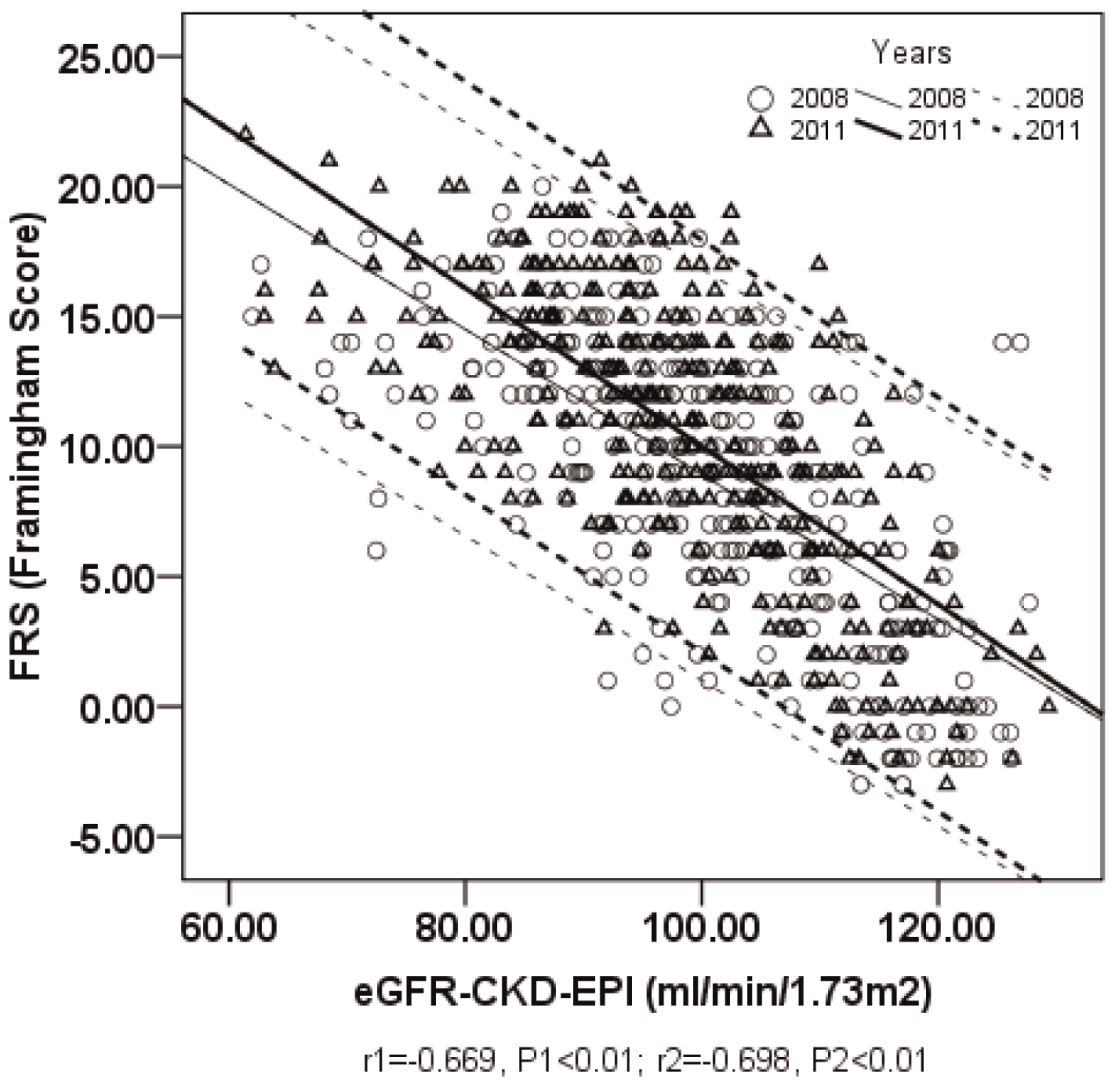
The relationship between FRS and eGFR_CKD-EPI_ in all participants. X-axis represents eGFR_CKD-EPI_ (estimated glomerular filtration rate calculated by CKD-EPI equation); Y-axis represents FRS (Framingham global CVD risk score); r1 and r2 represents the Pearson correlation coefficient between FRS and eGFR_CKD-EPI_ calculated in 2008 and in 2011; P1 and P2 represents *P* value of 2008 and 2011; thin straight line and thin dotted lines represent the regression line and its 95% confidence interval of 2008; bold straight line and bold dotted lines represent the regression line and its 95% confidence interval of 2011.

**Figure 2:**
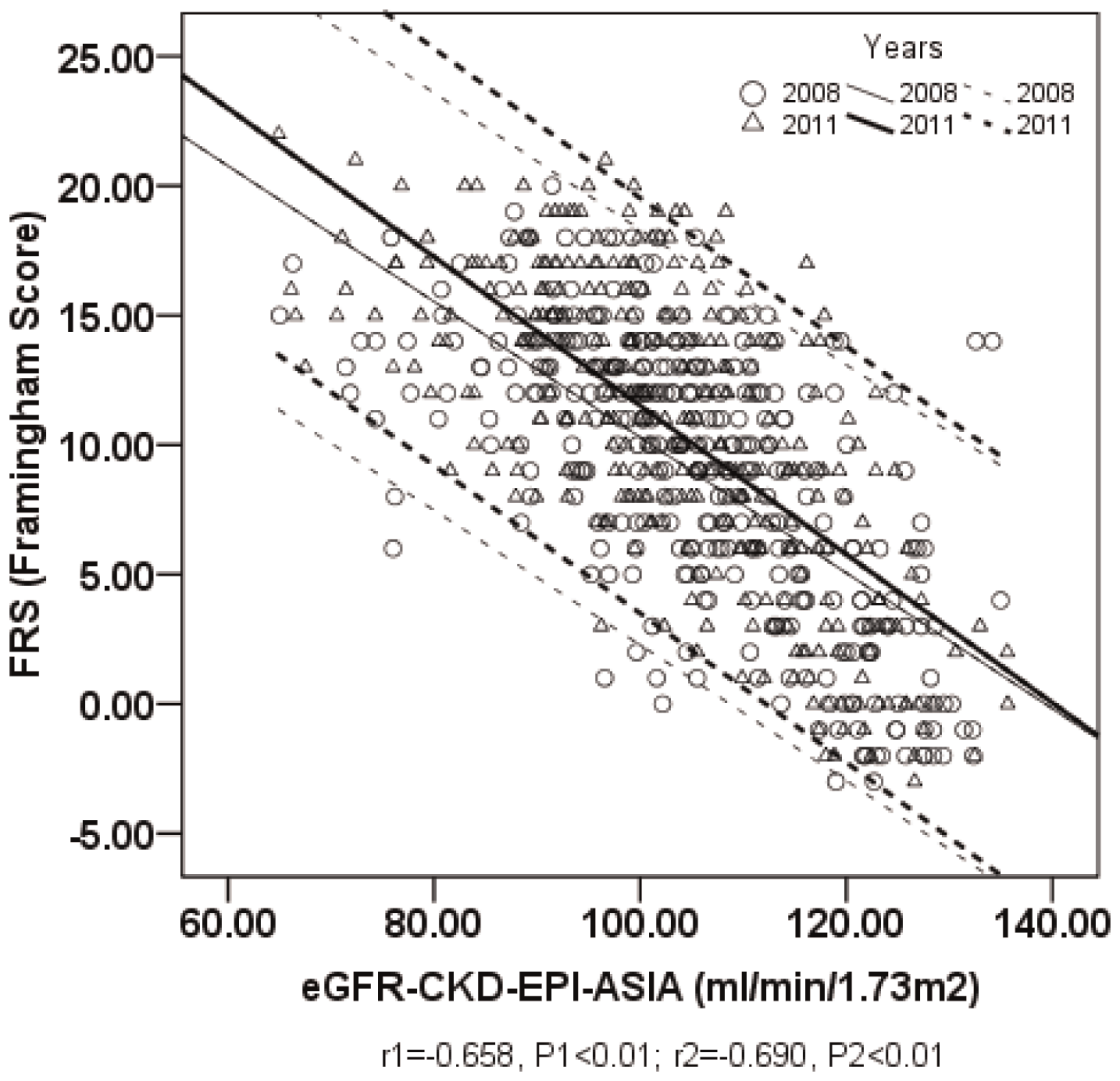
The relationship between FRS and eGFR_CKD-EPI-ASIA_ in all participants. X-axis represents eGFR_CKD-EPI-ASIA_ (estimated glomerular filtration rate calculated by CKD-EPI-ASIA); Y-axis represents FRS (Framingham global CVD risk score); r1 and r2 represents the Pearson correlation coefficient between FRS and eGFR_CKD-EPI-ASIA_ calculated in 2008 and in 2011; P1 and P2 represents *P* value of 2008 and 2011; thin straight line and thin dotted lines represent the regression line and its 95% confidence interval of 2008; bold straight line and bold dotted lines represent the regression line and its 95% confidence interval of 2011.

In 2008, significant inverse correlations between eGFR and FRS were confirmed with correlation coefficients of –0.669 (eGFR_CKD-EPI_, *P*<0.01) and –0.658 (eGFR_CKD-EPI-ASIA_, *P*<0.01) (Table 3, Figures 1 and 2). Similar results occurred in the low- and moderate-risk groups, with correlation coefficients of –0.615 (eGFR_CKD-EPI_, *P*<0.01), –0.609 (eGFR_CKD-EPI-ASIA_, *P*<0.01) and –0.282 (eGFR_CKD-EPI_, *P*<0.01), –0.285 (eGFR_CKD-EPI-ASIA_, *P*<0.01), respectively. However, there was no significant correlation between FRS and eGFR in the high-risk group.

**Table 3:** The relationship between FRS and eGFR evaluated in 2008 and in 2011.

FRS	Total (n = 333)	Low risk group (n = 153)	Moderate risk group (n = 150)	High risk group (n = 30)
eGFR_CKD-EPI_				
2008	−0.669*	−0.615*	−0.282*	−0.197
2011	−0.698*	−0.716*	−0.252*	−0.431#
eGFR_CKD-EPI-ASIA_				
2008	−0.658*	−0.609*	−0.285*	−0.197
2011	−0.690*	−0.712*	−0.253*	−0.431#

The relationship between FRS and eGFR was tested by the Pearson correlation coefficient method.

* *P*<0.01;

# *P*<0.05.

eGFR_CKD-EPI_: estimated glomerular filtration rate calculated by Chronic Kidney Disease Epidemiology Collaboration (CKD-EPI) equation; eGFR_CKD-EPI-ASIA_: estimated glomerular filtration rate calculated by Modified CKD-EPI equation for Asians.

Following a three-year follow-up, significant inverse correlations between FRS and eGFR were again confirmed in 2011, with correlation coefficients of –0.698 (eGFR_CKD-EPI_, *P*<0.01) and –0.690 (eGFR_CKD-EPI-ASIA_, *P*<0.01). In the low- or moderate-risk groups, significant inverse correlations were observed between FRS and eGFR, with correlation coefficients of –0.716 or –0.252 (eGFR_CKD-EPI_, *P*<0.01) and –0.712 or –0.253 (eGFR_CKD-EPI-ASIA_, *P*<0.01), respectively. In contrast to the lack of statistical significance between FRS and eGFR in the high-risk group in 2008, significant inverse correlations were obtained in 2011, with correlation coefficients of –0.431 (eGFR_CKD-EPI_ and eGFR_CKD-EPI-ASIA_, *P*<0.05).

Because the stratification of participants into a given risk group in 2011 was confounded by the other two groups, new groups containing the participants who were stratified into the same risk group in the two analyses were divided to estimate the association between eGFR and FRS. Table 4 shows confirmation of inverse correlations in the low-risk new group with correlation coefficients of –0.561 (eGFR_CKD-EPI_, *P*<0.01) and –0.557 (eGFR_CKD-EPI-ASIA_, *P*<0.01) in 2008 and –0.624 (eGFR_CKD-EPI_, *P*<0.01) and –0.622 (eGFR_CKD-EPI-ASIA_, *P*<0.01) in 2011. In the moderate-risk new group, inverse correlations were confirmed with correlation coefficients of –0.321 (eGFR_CKD-EPI_, *P*<0.01) and –0.326 (eGFR_CKD-EPI-ASIA_, *P*<0.01) in 2008 and –0.318 (eGFR_CKD-EPI_, *P*<0.01) and –0.329 (eGFR_CKD-EPI-ASIA_, *P*<0.01) in 2011. There was no association between eGFR and FRS in the high-risk new group in 2008 or in 2011.

**Table 4:** The relationship between FRS and eGFR evaluated in 2008 and in 2011 of new groups.

FRS	Low risk new group (n = 110)	Moderate risk new group (n = 107)	High risk new group (n = 28)
eGFR_CKD-EPI_			
2008	−0.561*	−0.321*	−0.049
2011	−0.624*	−0.318*	−0.268
eGFR_CKD-EPI-ASIA_			
2008	−0.557*	−0.326*	−0.049
2011	−0.622*	−0.329*	−0.268

The relationship between FRS and eGFR was tested by the Pearson correlation coefficient method.

* *P*<0.01;

# *P*<0.05.

eGFR_CKD-EPI_: estimated glomerular filtration rate calculated by Chronic Kidney Disease Epidemiology Collaboration (CKD-EPI) equation; eGFR_CKD-EPI-ASIA_: estimated glomerular filtration rate calculated by Modified CKD-EPI equation for Asians.

### 4. Correction for Traditional Cardiovascular Risk Factors

Multiple stepwise regression analyses were used to adjust for confounding factors, such as age, sex, smoking, SBP, TC, HDL-C and FBG, which were also considered as traditional cardiovascular risk factors. In the analysis conducted in 2008, following adjustment for sex, smoking, SBP, TC, HDL-C and FBG, the inverse association between eGFR and FRS was confirmed (*P*<0.001); however, when age was added to the list of confounding factors, the inverse association was no longer statistically significant (*P*>0.05). In contrast, with increasing FRS and decreasing eGFR, the inverse association between FRS and eGFR was statistically significant following adjustment for full traditional cardiovascular risk factors in the analysis conducted in 2011 (*P*<0.05).

## Discussion

Kidney function declines by approximately 10 ml/min/1.73 m^2^ per decade of life, even in the absence of CKD [11], which is defined as kidney damage or glomerular filtration rate (GFR) <60 ml/min/1.73 m^2^ for three months or more irrespective of cause [12]. As a result, a diagnosis of CKD in a healthy elderly individual with a GFR between 50 and 60 ml/min/1.73 m^2^ could be inaccurate [7]. Some studies have shown this age-related decline in kidney function even among those without kidney disease risk factors (i.e., hypertension, lower urinary tract disease) [13]. It is widely accepted that patients with chronic renal insufficiency appear to have a higher risk of CVD, independent of traditional cardiovascular risk factors [14]. However, the level of GFR at which CVD risk significantly increases is less clear. Contributing reasons for this uncertainty may include the controversial definition of renal insufficiency in elderly individuals or the replacement of other measurements of renal function with eGFR. However, the potential major causes for this uncertainty require further investigation.

Prior studies in high-risk populations suggest that the level of kidney function is an independent risk factor for CVD [15]–[18], although the strength of this suggestion in low-risk populations has been less conclusive [19]–[22]. Alternatively, some studies suggest that even small reductions in GFR within the apparently normal range are associated with increased risk of cardiovascular events, while others show that the increase in risk may not become apparent until the GFR declines to <60 ml/min/1.73 m^2^ [7]. Recently, a U-shaped relationship was observed between eGFR and CVD events, which makes it more difficult to interpret the eGFR threshold for increased risk [23].

The results of our two analyses both demonstrated that eGFR was inversely associated with FRS, and the lower limit of eGFR was not 60 ml/min/1.73 m^2^. Unfortunately, we have no precise knowledge about the cut-off point for eGFR at which CVD risk significantly increases. As we extracted a database from a healthy population, the results derived in 2008 suggest that in healthy populations, the inverse correlation between eGFR and FRS was gradually attenuated from the low- to high-risk groups. However, this association was influenced by traditional cardiovascular risk factors. The results of multiple stepwise regression analysis suggested that age is the major confounding factor contributing to increased FRS and decreased eGFR. This means that in healthy populations, age is the major risk factor for CVD.

As Table 3 shows, the inverse association between eGFR and FRS became stronger with increasing FRS in the entire study population and in the low- and high-risk groups. However, conflicting conclusions were reached in the moderate-risk group with correlation coefficients of –0.282 (eGFR_CKD-EPI_, *P*<0.01) and –0.285 (eGFR_CKD-EPI-ASIA_, *P*<0.01) in 2008 and –0.252 (eGFR_CKD-EPI_, *P*<0.01) and –0.253 (eGFR_CKD-EPI-ASIA_, *P*<0.01) in 2011. The cause of this is that the risk stratification was based on FRS calculated in 2008, which confounds that risk stratification by the other two in obtained in 2011. For example, there were 153 participants stratified into the moderate-risk group in 2008; however, based on FRS calculated in 2011, there were only 107 participants divided into the moderate-risk group, four participants divided into the low-risk group, and 39 participants divided into high-risk group. As the strength of association gradually decreased from the low- to high-risk groups, the association in the moderate-risk group may attenuate because of confounding by the increasing number of high-risk participants. However, it is unconfirmed whether the result of the high-risk group is confounded by two participants being stratified into the moderate-risk group.

To eliminate confounding factors, new groups of participants who were stratified into the same risk group were constituted for the two analyses. As shown in Table 4, in the moderate-risk new group, the inverse association became stronger with increasing FRS with correlation coefficients of –0.326 (eGFR_CKD-EPI-ASIA_, *P*<0.01) in 2008 and –0.329 (eGFR_CKD-EPI-ASIA_, *P*<0.01) in 2011. Because this study was conducted in a Chinese population, the conclusion drawn from CKD-EPI-ASIA equation is considered to be more reliable. There was no association between eGFR and FRS in the high-risk new group in the two analyses. Thus, it is confirmed that the results in the high-risk group were confounded by participants divided into the moderate-risk group. However, it does not mean we can confirm there are no associations between eGFR and FRS in the high-risk group, partially because there were too few participants to draw this conclusion. Another reason is insufficiencies in the calculation of FRS. As we know, if patients are older than 75 years, this will produce an underestimation of FRS and influence the results. A conclusion was drawn that the inverse relationship between eGFR and FRS became stronger with increasing FRS in the same risk group stratification.

Another conflicting conclusion occurred in the multiple stepwise regression results. In contrast to the previous analysis, eGFR was shown to be a risk factor for FRS in the second analysis, being independent of traditional cardiovascular risk factors. The reason for this phenomenon is the change in study participants. There was no diagnosed CVD following three-year follow-up, but some participants were adversely affected with comorbid conditions, such as hypertension or diabetes. Thus, it is inappropriate to evaluate the association between eGFR and FRS without considering comorbid conditions. Our results suggested that eGFR had a different effect on predicting CVD risk in different populations.

There are several potential explanations as to why the level of kidney function may be an independent risk factor for increased risk of CVD events [11]. First, and somewhat related, the level of kidney function may be a measure of residual confounding from traditional CVD risk factors [24]. Second, a decrease in the level of kidney function may be associated with increased levels of nontraditional CVD risk factors, such as elevated homocysteine levels, oxidative stress, inflammatory mediators, and remnant cholesterol particles, which were not measured in the study and, therefore, not adjusted for in our analysis [11]. Third, the level of kidney function may be a marker of undiagnosed vascular disease or alternatively a marker for the severity of diagnosed vascular disease. Finally, reduced kidney function itself may be a risk factor for progression of ventricular remodeling and cardiac dysfunction; that is, kidney disease may alter sodium and fluid handling and thereby alter cardiac function. Unfortunately, our retrospective analyses cannot differentiate among these four possibilities [24].

The search for novel markers to better predict cardiovascular disease in patients is of considerable clinical interest as it may lead to improved primary prevention. A population-based study in four U.S. communities, followed for approximately ten years, demonstrated that higher levels of cystatin C, *β*_2_-microglobulin (B2M) and *β*-trace protein (BTP) are also predictors of CVD events. They concluded that B2M and BTP levels share advantages of cystatin C over eGFR_CKD-EPI_ in predicting CVD outcomes. These additional markers may be helpful in improving estimation of risk associated with decreased kidney function beyond current estimates based on eGFR_CKD-EPI_ [25]. A study performed in a community-based Chinese population showed that low eGFR is associated with detectable high-sensitivity cardiac troponin T (hs-cTnT). Moreover, eGFR and high predicted Framingham coronary heart disease risk are associated with detectable hs-cTnT in subjects with moderate-to-severe reduced renal function [26]. Urinary albumin-creatinine ratio has also been examined as a predictor of CVD, although Clase et al. [27] concluded that urinary albumin-creatinine ratio adds very little to traditional cardiovascular risk factors in patients with high cardiovascular risk. In the near future, perhaps, conflicting conclusions may be obtained in different populations and with different methods. Recently, increasing attention has focused on relationships between changes in gene expression and CVD. Eyster et al. [28] reported that the gene expression pattern in arteries containing atherosclerotic plaques is profoundly different from that of relatively unaffected arteries. Cytokine gene polymorphisms [29] and impact of inflammation on epigenetic DNA methylation [30] are also considered as novel risk factors for CVD. Most previously published studies have been conducted in CKD patients. In the future, we will try to assess associations between gene expression as well as other novel biochemical parameters and CVD to indentify biomarkers for CVD in subjects with eGFR above 60 ml/min/1.73 m^2^, which allows CVD risk analysis to be a routine laboratory procedure. Additionally, while more than 99% of the participants in the present study were from the Han Yellow race, further study will contain populations comprising subjects from other races so that race and ethnic culture can be considered as potential factors for CVD development.

Our study has several potential limitations that merit comment. First, our population only contained 333 subjects. Although well-represented across a strata of age, sex, and cardiovascular risk, larger samples are necessary to fully elucidate the association between FRS and eGFR. Second, we may have underestimated FRS because the definition of diabetes in our study was only based on fasting blood glucose. If the postprandial blood glucose (PBG) for a participant reached the level of diabetes but FBG was in the normal range, his diagnosis of diabetes would be missed. Furthermore, the Framingham risk equations used in this study may not be as well validated in Chinese participants, especially in the elderly, as mentioned above. Third, there were a larger number of females (81.7%) compared to males (18.3%) in the low-risk group, but sex hormones changes in females were not considered during blood pressure measurements. Many studies have confirmed that blood pressure is higher in males than in females at similar ages [31]–[34]. The hypotensive effects of estradiol in females are well-recognized. During the luteal phase of the menstrual cycle (when estradiol level peaks), blood pressure is lower than during the follicular phase [35], [36]. Although we calculated that 90 women were pre-menopausal and 35 women were post-menopausal, we did not record which phase of the menstrual cycle the pre-menopausal women were in and did not measure levels of sex hormones. Fourth, the use of eGFR to assess renal function in healthy individuals could be prone to measurement bias [17]. Unfortunately, there is no precise measure of renal function that is presently routinely used in large epidemiologic studies. Inulin or iothalamate clearances are invasive and cumbersome, and 24-h urine collections are very unreliable [37]. Although the more recently developed equations have the widest acceptance, a recent Chinese study demonstrated that two CKD-EPI equations seemed to estimate a reasonable distribution of eGFR in healthy Chinese adult populations compared with two MDRD equations (MDRD and IDMS-MDRD equations) [10]. Nevertheless, we cannot exclude a slight possibility of measurement bias. Fifth, we did not have information with regard to proteinuria and albuminuria because we did not collect urine samples. Recent studies have shown that albuminuria, an alternate marker for the presence of kidney disease, may be an independent risk factor for CVD outcomes [20]. Unfortunately, we did not obtain levels of albumin. Similar markers include cystatin C, other CVD risk factors and non-traditional cardiovascular risk factors. Sixth, because of the relatively short follow-up time, we did not yet find diagnosed CVD and could not calculate rates of cardiovascular events in our study. These results should be confirmed by long-term observations.

## Supporting Information

Table S1 STROBE Statement–Checklist of items that should be included in reports of *cohort studies*.
(DOC)
